# Evaluation of baseline optic disc pit and optic disc coloboma maculopathy features by spectral domain optical coherence tomography

**DOI:** 10.1186/s40942-023-00484-7

**Published:** 2023-08-07

**Authors:** Vishma Prabhu, Rubble Mangla, Isha Acharya, Ashit Handa, Atul Thadani, Yash Parmar, Naresh Kumar Yadav, Jay Chhablani, Ramesh Venkatesh

**Affiliations:** 1 Dept. of Retina and Vitreous, #121/C, Chord Road, 1st R block Rajaji Nagar, Bangalore, 560010 India; 2 Medical Retina and Vitreoretinal Surgery, University of Pittsburgh School of Medicine, 203 Lothrop Street, Suite 800, Pittsburgh, PA 15213 USA

**Keywords:** Optic disc pit, Optic disc coloboma, Optical coherence tomography, Baseline findings

## Abstract

**Purpose:**

The aim of this study is to describe and compare the baseline demographic, ocular, and imaging characteristics of a cohort of patients with optic disc pit (ODP) or optic disc coloboma (ODC) maculopathy.

**Methods:**

This retrospective study included patients diagnosed with ODP or ODC on clinical examination between June 2017 and December 2022. These patients’ baseline demographics, ocular characteristics, and optical coherence tomography (OCT) imaging characteristics were analyzed.

**Results:**

Fundus examination revealed 11 eyes of 11 patients with ODP and 14 eyes of 9 patients with ODC, respectively. On OCT, maculopathy was observed more frequently in ODP (n = 10) than in ODC (n = 4) [p = 0.004] cases. Eyes with ODP were more likely to exhibit retinoschisis and/or serous macular detachment [SMD] (n = 7, 70%), communication of the retinoschisis with the optic disc (p = 0.015), whereas the SMD did not communicate with the optic disc (p = 0.005), and significant outer retinal layer thinning (p = 0.015). In contrast, eyes with ODC exhibited only SMD (p = 0.005) and no retinoschisis on the non-colobomatous retina. SMD in ODC communicated with the margin of the optic disc. In both clinical entities, hyperreflective foci were observed in the SMD.

**Conclusion:**

In summary, baseline maculopathy characteristics on OCT, including its type, location, and relationship to the optic disc, are among the most distinguishing characteristics between an ODP and an ODC.

**Trial Registration Number:**

Not applicable.

**Supplementary Information:**

The online version contains supplementary material available at 10.1186/s40942-023-00484-7.

## Introduction

Excavated congenital optic disc anomalies include megalopapilla, peripapillary staphyloma, optic disc coloboma (ODC), optic disc pit (ODP), and morning glory disk anomaly (MGDA) [1]. ODC, ODP and MGDA share a common embryological origin, exhibit similar optic disc morphology, remain stationary, and are frequently associated with serous retinal detachments [2, 3]. The most common explanation regarding the formation of congenital cavitary optic disc entities proposes that they arise from either the incomplete closure of the fetal fissure during embryogenesis or an impaired differentiation of the peripapillary sclera from the primary mesenchyme [4]. Despite sharing a similar embryonic origin, these entities exhibit distinct visual characteristics when observed at the optic disc. An ODP refers to a central depression resembling a crater in the optic disc, where the typical tissue of the optic nerve is absent. This condition is predominantly found on the temporal aspect of the optic disc [5]. An ODC is distinguished by a concave excavation, typically with well-defined boundaries, located inferiorly. It lacks a central glial tuft and typically exhibits normal vasculature within the optic disc [5]. In contrast, the primary ophthalmoscopic characteristics of a MGDA consist of a conical depression with a tuft of glial tissue located at the center of the depression, accompanied by a pronounced and increased retinal vascularity extending outward from the outer margins of the optic disc [5, 6]. Congenital ODPs are seen alone or occasionally in combination with ODCs [7, 8]. These observations suggest that congenital ODPs may be pathologically related to ODCs.

In terms of vision, patients with ODPs and ODCs typically exhibit excellent vision, unless further complicated by retinal schisis (splitting) and serous macular detachment (SMD) [9]. On the other hand, MGDA is associated with an increased risk of retinal detachment and poor visual acuity [9]. The only reason to consider treatment/intervention in cases of ODP or ODC is the development of SMD [10]. Treatment/intervention is generally considered for cases demonstrating new macular serous detachment with recent onset visual symptoms or cases with progressive increase in the SMDs [10–12]. A SMD affects approximately two-thirds of patients with congenital ODPs [13]. Optical coherence tomography (OCT) has revealed that retinoschisis-like retinal separations occur frequently during the development of SMDs in eyes with congenital ODPs [14]. In eyes with ODC, similar SMDs with retinoschisis-like separations have been reported [15, 16]. The exact etiology of macular detachments caused by ODPs or ODCs is still unknown. Furthermore, the pathogenesis for developing maculopathy in these embryologically similar entities could be significantly different.

In order to conduct a more in-depth investigation, we aimed to compare and report the baseline OCT findings in eyes with ODP and ODC maculopathy, as well as provide a plausible theory for its development in these cases.

## Methods

Between June 2017 and December 2022, cases diagnosed with congenital ODP and ODC at a tertiary eye hospital were included in this retrospective observational study. A typical ODP is a solitary, ovoid, grey-white crater-like excavation of the optic disc, usually at its temporal margin. The ODC is distinguished by a bowl-shaped excavation that is often inferiorly located and has sharp borders. According to Ida-Mann’s classification, this is a type 4 coloboma [17]. Other congenital optic disc cavitary anomalies such as MGDA, Pedlar’s coloboma, megalopapilla and peripapillary staphyloma and other acquired excavated optic disc pathologies such as avulsed optic nerve following trauma were excluded from the study.

All of these cases’ medical records were reviewed, and demographic and ophthalmic data were compiled. Age, gender, laterality of involvement, visual acuity, spherical equivalent, intraocular pressure of both the study and the fellow eye, and the presence of additional choroidal coloboma were all recorded. The visual acuity was initially noted in Snellen’s format and was later converted to logarithm of minimum angle of resolution (logMAR) for statistical purpose. On OCT scans obtained with the Spectralis (Heidelberg Engineering, Germany) machine, the presence of maculopathy following an ODP or ODC was confirmed. The horizontal line raster OCT scans were obtained using the enhanced depth imaging mode passing through the optic nerve head and macular region. On OCT, maculopathy secondary to ODP or ODC was identified by the presence of retinoschisis and/or SMD. Other features of maculopathy that were observed included communication of the retinoschisis and/or SMD with the ODP or ODC, the presence of outer retinal layer thinning with increased visibility of the underlying choroidal structures, and hyperreflective clumps within the SMD.

### Statistical tests

All data were analysed using GraphPad Prism version 9.5.0 (730) for Windows, GraphPad Software, San Diego, California USA, www.graphpad.com. The vision data at presentation was documented as Snellen’s vision data and was converted to logarithm for minimum angle of resolution for analytical purpose. Only statistical tests related to the analysis of non-parametric data were used in this study. Quantitative data between the 2 groups of cases were analysed using the Mann-Whitney U test. Chi-square test was used to compare the categorical data between 2 groups. P values < 0.05 were considered statistically significant.

## Results

**Fig. 1:**
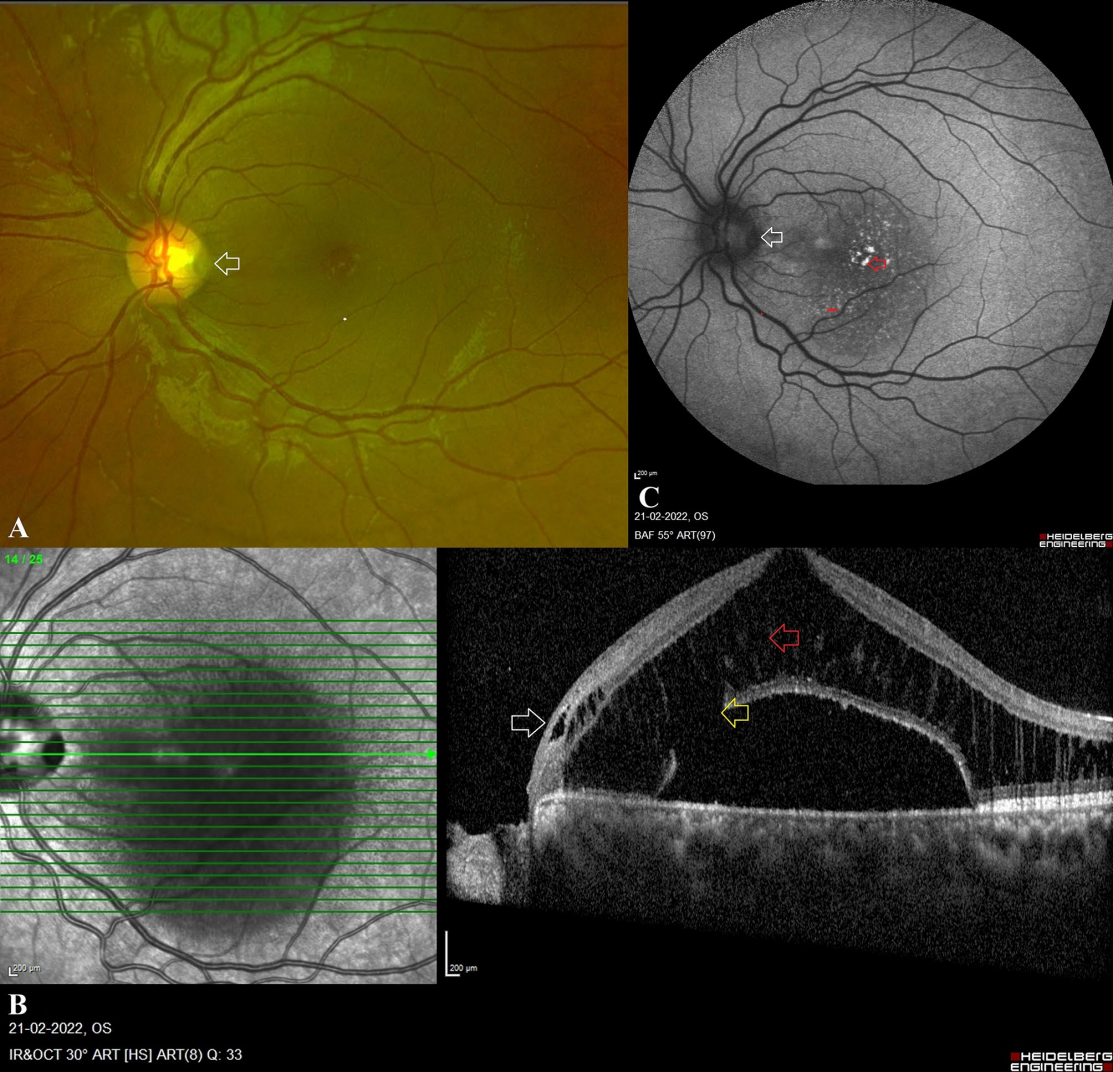
A case of Optic disc pit with maculopathy: **A**: This cropped colour fundus image (Optos, Daytona, UK) belongs to a 26-year-old female showing a grey-white translucent defect at the temporal portion of the optic disc suggestive of optic disc pit (white arrow). Her presenting visual acuity was 20/80, N12 in the affected left eye. **B**: The optical coherence tomography scan section through the ODP and macula shows the temporal defect suggestive of an ODP with schisis at the nerve fibre layer and at the junction of inner nuclear layer – outer plexiform layers (white arrow), and schitic fluid further dissecting the outer nuclear layer of the retina (red arrow) followed by a focal outer retinal defect (yellow arrow) causing serous macular detachment (SMD). There is no communication of the SMD with the optic disc pit. **C**: Fundus autofluoroscent image shows the hypoautofluorescent ODP (white arrow) at the temporal portion of the optic disc with a number of hyper autofluoroscent spots (red arrow) at the posterior pole indicative of shaggy photoreceptors and a long-standing SMD

**Fig. 2:**
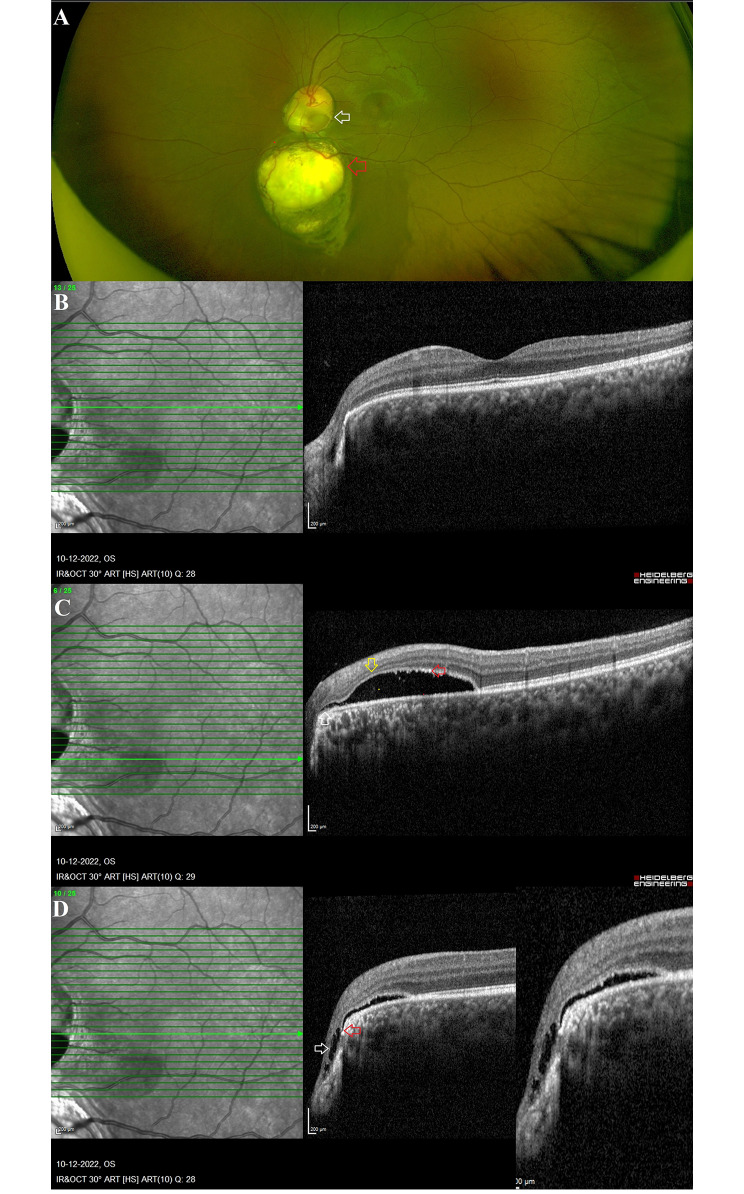
A case of optic disc coloboma with maculopathy: **A**: Cropped Optos image of the posterior pole of a 24-year-old girl shows a bowl-shaped inferior excavation with well-defined borders (white arrow) suggestive of optic disc coloboma with an additional choroidal coloboma inferior to it (red arrow). **B**: OCT scan image passing through the fovea shows a normal foveal contour without subfoveal subretinal fluid. **C**: OCT scan image passing through the serous retinal detachment. The serous retinal detachment is seen communicating directly with the optic disc coloboma (white arrow). Outer nuclear layer thinning is noted (yellow arrow) with multiple hyperreflective dots noted within and on the under surface of the neurosensory detachment (red arrow). **D**: OCT scan through the optic disc coloboma margin shows the schitic marginal intercalary membrane (white arrow) and the communication between the sub intercalary membrane space inside the coloboma and the subretinal space outside the coloboma (red arrow)

Eleven eyes of eleven patients with ODP and fourteen eyes of nine patients with ODC were included in this study. These anomalies were identified in 15 patients who presented with diminished vision while in the remaining 5 cases, the ODPs and ODCs were identified incidentally during ocular examination screening. In Table 1, comparisons between demographic and ocular characteristics are described in detail. Patients with ODC presented to the clinic at a younger age than those with ODP (p = 0.02). ODC eyes exhibited bilateral involvement (n = 6, 67%), and both the study eyes and fellow eyes exhibited the presence of additional choroidal coloboma. The spherical equivalents of the study eye and fellow eye were comparable between ODP and ODC cases. The visual acuity of the fellow eye was significantly lower in the ODC group (p < 0.05), whereas the visual acuity of the study eye was comparable between the two groups. In the ODP (n = 10) group, maculopathy was more prevalent than in the ODC (n = 4) group (p < 0.05) **[**Figs. 1 and 2**]**. Table 2 details maculopathy-related findings in both groups. 70% (n = 7) of eyes with ODP-maculopathy exhibited retinoschisis and SMD. Communication of the retinoschisis was observed in 8 (80%) eyes and thinning of the outer retinal layer was more pronounced in 8 (80%) cases of ODP. One eye with ODP showed the presence of SMD alone. In contrast, SMD was the most common maculopathy finding in eyes with ODC. No patient presented with retinoschisis outside the coloboma. Communication between the SMD and ODC was observed in each of the four eyes with ODC maculopathy. In both groups, hyperreflective clumps were observed within the SMD (p > 0.05).

**Table 1:** Demographic data comparisons between optic disc pit and optic disc coloboma

	Optic disc pit	Optic disc coloboma	P value
No. of eyes/patients	11/11	14/9	
Mean age (years) [mean, SD]	30.73 ± 9.676	20.86 ± 11.31	0.02
Visual symptoms at presentation	11 (100)	4 (44)	0.008
Gender distribution (M: F)	5:6	3:6	0.67
Bilateral involvement (n, %)	0 (0)	6 (67)	0.002
Laterality (RE: LE)	5:6	7:7	> 0.999
No. of study eyes with choroidal coloboma (n, %)	0 (0)	6 (43)	0.02
No. of fellow eyes with choroidal coloboma (n, %)	0 (0)	5 (36)	0.046
Spherical equivalent study eye (D)	-0.466 ± 1.293	-0.375 ± 1.313	0.764
Spherical equivalent fellow eye (D)	-0.716 ± 1.537	-0.500 ± 1.146	0.988
Mean logMAR visual acuity study eye [mean, SD], (Snellen equivalent)	0.589 ± 0.478 (20/78)	0.531 ± 0.417 (20/68)	0.798
Mean logMAR visual acuity fellow eye [mean, SD], (Snellen equivalent)	0.016 ± 0.054 (20/21)	0.59 ± 0.566 (20/78)	0.001
IOP study eye (mm Hg) [mean, SD]	14.27 ± 2.611	14.64 ± 4.217	0.925
IOP fellow eye (mm Hg) [mean, SD]	14.45 ± 3.11	14.93 ± 4.615	0.945

Abbreviations: RE – right eye; LE – left eye; logMAR – logarithm of minimum angle of resolution; IOP – intraocular pressure; SD – standard deviation; D - diopters

**Table 2:** OCT findings in patients with optic disc pit and optic disc coloboma:

	Optic disc pit (n = 11)	Optic disc coloboma (n = 14)	P value
	Maculopathy (n = 10)	Maculopathy (n = 4)	0.004
RS alone (n, %)	2 (20)	0 (0)	> 0.999
SMD alone (n, %)	1 (10)	4 (100)	0.005
RS + SMD (n, %)	7 (70)	0 (0)	0.07
Communication of RS with optic disc (n, %)	8 (80)	0 (0)	0.015
Communication of SMD with optic disc (n, %)	1 (10)	4 (100)	0.005
Outer retinal layer thinning (n, %)	8 (80)	2 (0)	0.015
Hyperreflective clumps within the SMD (n, %)	8 (80)	2 (50)	0.521

Abbreviations: RS – retinoschisis; SMD – serous macular detachment

## Discussion

This study showed significant differences between ODP and ODC maculopathy features on OCT. Greater prevalence of maculopathy, presence of retinoschisis and SMD, and continuation of retinoschisis but not SMD with the optic disc were observed in eyes with ODP, whereas eyes with ODC exhibited a lower prevalence of maculopathy, presence of SMD without retinoschisis outside the coloboma, and communication of the SMD with the ODC. In addition, there were differences between the two groups in terms of demographics and ocular characteristics.

The optic nerve head is composed of retinal ganglion cell axons, blood vessels, glia, and connective tissue. The optic disc is widely regarded as the central location of impairment in congenital cavitary defects such as ODP, ODC, MGDA and megalopapilla and in acquired conditions like glaucoma and peripapillary staphyloma [5, 18, 19]. The observed disparities in our study’s findings can be attributed to different gross and histological anatomical characteristics, variations in lesion margins, and differing mechanisms underlying the onset of maculopathy in these two entities.

Congenital optic pits commonly affect the temporal optic disc, although they have the potential to occur in various other areas. Histologically, optic pits are distinguished by the presence of dysplastic retina herniations into the subarachnoid space, often occurring through a major defect in the lamina cribrosa [20]. On the other hand, an ODC is a distinct, bright white, concave depression located in the lower portion of an enlarged optic disc, with a size that is notably larger than that of an ODP [21]. Both of these papillary defects have the potential for communication between various spaces, including the vitreous space, subarachnoid space, intraretinal space, subretinal space, and orbital spaces, either directly or indirectly. Therefore, an ODP is often classified as an “unusual and small coloboma” located at the optic nerve head.

Our study yielded several interesting and highly significant findings when examining the demographic and ocular characteristics of these two clinical entities. The patients diagnosed with ODP exhibited a higher mean age and a greater prevalence of visual symptoms upon their initial presentation at the clinic, in comparison to patients diagnosed with ODC. Patients with ODP commonly experience visual difficulties when maculopathy develops. Maculopathy typically manifests during the period spanning the second to fourth decades of an individual’s life within the context of ODP [22]. Maculopathy that arises as a consequence of ODC also manifests at a similar time. However, owing to the large size of the defect and its greater likelihood of being associated with choroidal coloboma, the visual symptoms of ODC become apparent at a notably earlier stage, leading to an earlier manifestation in clinical settings [7]. In addition, it was observed that the visual acuity of the fellow eyes in individuals with ODC was notably inferior compared to the visual acuity of the eyes with ODP. This phenomenon may also be attributed to the presence of choroidal colobomas in both eyes among individuals with ODC. Patients with ODC tend to present at a relatively early age to the clinic due to the manifestation of poor vision in the fellow eye and the accidental discovery of ODC in the affected eye. Ohno-Matsui et al. reported similar findings pertaining to the occurrence of choroidal coloboma in cases of ODC [23].

Both of these clinical conditions commonly exhibit maculopathy, which is typically characterized by the presence of intraretinal fluid/retinoschisis or SMD. A higher prevalence of maculopathy symptoms was observed in the group of individuals with ODP. One potential factor contributing to the observed phenomenon in the current study is the comparatively earlier onset of ODC cases, which may result in the gradual development of maculopathy over a period of time. The structure of the optic disc’s margin may also serve as a potential barrier against the development of maculopathy during the initial phases. The classification system proposed by Ida-Mann categorizes an ODC as a choroidal coloboma of type IV [17]. Hence, the anatomical characteristics of the margin in an ODC will exhibit similarities to the margin anatomy observed in choroidal colobomas located in other regions of the fundus. The intercalary membrane (ICM) is a continuation of the inner retina from the non-colobomatous area that extends over the colobomatous area. This conversion of the inner retina into an ICM may be transient or abrupt at the coloboma margin [24]. Several studies utilizing swept source OCT have demonstrated that the occurrence of subclinical or clinical retinal detachments (RDs) is influenced upon the presence of microbreaks at the marginal ICM, which are caused by the continuous traction exerted by the vitreous at the coloboma margin [25, 26]. The development of these marginal ICM breaks may require a significant amount of time, which could explain the lack of visibility of maculopathy characteristics in ODC cases in our study.

In our study, we observed that the maculopathy characteristics of SMD or inner retinal schisis presence, location, communication with the papillary defect and focal outer retinal layer defects were distinct between the two entities. We observed that eyes with ODP were more likely to exhibit simultaneous inner retinal schisis and SMD. In contrast, eyes with ODC exhibited only SMD in every case, and no patient exhibited retinoschisis outside the coloboma. In addition, we observed that the inner retinal schisis communicated with the optic disc defect in eyes with ODP, whereas the SMD failed to communicate with the ODP in most cases. However, the SMD communicated with ODC in all four eyes. These intriguing differences could be explained by the pathophysiology underlying the development of maculopathy in both of these entities. In an ODP maculopathy, regardless of the source of fluid, Lincoff et al. have described a generally accepted sequence of retinal fluid accumulation and progression of its formation [11, 27, 28]. The fluid originating from the ODP initially induces an inner retinal separation resembling schisis, subsequently leading to the development of an outer layer macular hole beneath the inner layer. Subsequently, the fluid proceeds to dissect the subretinal region, resulting in the detachment of the outer retina. Additionally, it has been reported that nearly all cases of ODP maculopathy exhibit intraretinal fluid within the outer nuclear layer, while none exhibit solely subretinal fluid. This observation provides support to the hypothesis that the fluid initially infiltrates the inner retinal layers before progressing to the subretinal area. [29]. It has been hypothesized that, as fluid accumulates intraretinally in eyes with ODP maculopathy, a pressure gradient forms, directing the fluid towards the outer retinal layers and then into the subretinal space [30]. In one eye, which showed the presence of SMD alone in a case of ODP, the possible explanation could be a breakdown in communication between the ODP and inner retinal space as a result of a previous laser barrage to the optic disc margin. In an ODC, the vitreous’ persistent traction on the ICM at the coloboma margin causes schisis-like defects in both the marginal and central portions of the ICM. This persistent traction eventually causes micro or mini breaks at the marginal ICM, allowing fluid to enter the sub retinal space outside the coloboma, resulting in subclinical or clinical retinal detachment. Retinoschisis outside the coloboma without retinal detachment is uncommon in eyes with ODC, with the exception of eyes with long-standing retinal detachment, in which the retinal detachment itself causes degenerative splitting of the inner retinal layers. This best explains the glaring differences between ODP and ODC maculopathy characteristics noted in our study. In instances of ODP and ODC, hyperreflective dots are observed in the SMD. These are comparable to the shaggy photoreceptors observed in eyes with long-standing subretinal fluid, such as in chronic CSCR, autosomal recessive bestrophinopathy, and choroidal melanoma [31–35]. This finding also suggests that the subretinal fluid is thick, viscid and slowly resorbing, which explains why SMD absorption requires more time.

ODP and ODC share a similar embryological origin, resulting from incomplete closure of the fetal fissure’s proximal portion. The only difference is the size of the defect: the size of ODC defects is greater in comparison to ODP defects, with the latter being significantly smaller. As part of the choroidal coloboma spectrum, the ODC is frequently associated with other systemic syndromes, such as the CHARGE and COACH syndromes, whereas the ODP is typically sporadic and rarely associated with other systemic syndromes [36].

This study has clinical significance because the OCT characteristics and visual symptoms in ODP and ODC may serve as a guide when considering intervention in such cases. Observation, laser retinopexy, pneumatic retinopexy, and pars plana vitrectomy with internal limiting membrane peeling are among the various treatment modalities for ODC or ODP cases. We recommend that clinicians take a step-by-step approach when considering interventional treatment for ODP or OPC cases based on the presence of maculopathy, progression of maculopathy, and recent onset of visual symptoms. In addition, we stress the importance of analysing the entire cube scan at the optic disc margin in order to evaluate the communication between the SRF and ODP or ODC when planning intervention in these cases. Moreover, the role of swept-source OCT imaging cannot be overlooked in such clinical settings, particularly in eyes with ODC.

A few drawbacks exist in the present study. Despite the utilization of the enhanced depth imaging mode on the spectral domain Spectralis OCT machine, the acquisition of a comprehensive image encompassing the entirety of the subarachnoid space and optic nerve sheath remained challenging. The optimal approach for imaging and computation in this scenario would involve the utilization of swept-source OCT imaging. Obtaining clear OCT images proved to be a difficult task, especially in cases where eyes had ODC. Follow up changes, and treatment outcomes were not addressed. The primary objective of this study was to investigate the initial imaging disparities between eyes affected by ODP and ODC maculopathy. The aim was to offer plausible interpretations for these findings and to furnish clinicians with a helpful tool for devising treatment strategies.

In summary, there are obvious disparities in the baseline maculopathy characteristics observed on OCT between patients diagnosed with ODP and ODC. The primary distinguishing characteristics between the two clinical entities are the presence of retinoschisis and SMD, along with their specific location and relationship to the optic disc.

### Electronic supplementary material

Below is the link to the electronic supplementary material.

Supplementary Material 1

## Data Availability

The datasets used and/or analysed during the current study are available from the corresponding author on reasonable request.

## Declarations

### Animal research

“This article does not contain any studies with animals performed by any of the authors.”

## Abbreviations

ODC: Optic disc coloboma
ODP: Optic disc pit
MGDA: Morning glory disc anomaly
SMD: Serous macular detachment
OCT: Optical coherence tomography
ICM: Intercalary membrane
