# Supplementary material for: Evaluation of baseline optic disc pit and optic disc coloboma maculopathy features by spectral domain optical coherence tomography
Source: Int J Retina Vitreous. 2023 Aug 7;9:46. doi: 10.1186/s40942-023-00484-7 (PMC10405376; doi:10.1186/s40942-023-00484-7)
Supplement: Supplementary file 1 — Supplementary Material 1 [file 40942_2023_484_MOESM1_ESM.docx]

Reviewer reports:

Our system has found an overall similarity score of 26%, meaning your manuscript has sentences that are present in other publications, especially in the introduction and discussion. Please rephrase most of it before it can be accepted for publication.

Reply: Paraphrasing of the sentences in the introduction and discussion sections have been made. Similar appearing sentences have been modified so as to reduce the similarity score.
